# Supplementary material for: High-Grade B-Cell Lymphoma (HGBL) with MYC and BCL2 and/or BCL6 Rearrangements Is Predominantly BCL6-Rearranged and BCL6-Expressing in Taiwan
Source: Cancers (Basel). 2021 Mar 31;13(7):1620. doi: 10.3390/cancers13071620 (PMC8059140; doi:10.3390/cancers13071620)
Supplement: Supplementary file 1 [file cancers-13-01620-s001.pdf]

cancers

MDPI

Supplementary

High-Grade B-Cell Lymphoma (HGBL) with *MYC* and *BCL2* and/or *BCL6* Rearrangements Is Predominantly BCL6-Rearranged and BCL6-Expressing in Taiwan

Cheng-Chih Tsai et al.

Citation: Tsai, C.-C.; Su, Y.-C.; Bamodu, O.A.; Chen, B.-J.; **Tsai**, W.-C.; Cheng, W.-H.; Lee, C.-H.; Hsieh, S.-M.; Liu, M.-L.; Fang, C.-L.; et al. High-Grade B-Cell Lymphoma (HGBL) with *MYC* and *BCL2* and/or *BCL6* Rearrangements Is Predominantly BCL6-Rearranged and BCL6-Expressing in Taiwan. *Cancers* **2021**, *13*, 1620. https://doi.org/10.3390/cancers13071620

Academic Editor: Alexey Ushmorov; Chalid Assaf

Received: 15 January 2021
Accepted: 26 March 2021
Published: 31 March 2021

Publisher's Note: MDPI stays neutral with regard to jurisdictional claims in published maps and institutional affiliations.

Copyright: © 2021 by the author. Licensee MDPI, Basel, Switzerland. This article is an open access article distributed under the terms and conditions of the Creative Commons Attribution (CC BY) license (http://creativecommons.org/licenses/by/4.0/).

**Number of cases**		
**BCL6-rearrangement**	**n (%)**	
0	6 (31.6%)	
1	13 (68.4%)	
**BCL2-rearrangement**	**n (%)**	
0	9 (47.4%)	
1	10 (52.6%)	
**BCL6*BCL2**		
**BCL6-rearrangement**	**BCL2-rearrangement**	**n (%)**
0	0	0 (0.0%)
	1	6 (31.6%)
1	0	9 (47.4%)
	1	4 (21.0%)

**Variable - Outcome analysis**		
	**BCL6-rerrangement**	
Chi-squared	5.7918	
p-value	0.02	
**Hazard ratios with 95% Confidence Interval**		
**Factor**	**0**	**1**
0	-	0.2659 (0.04496 to 1.5720)
1	3.7613 (0.6361 to 22.2400)	-
	**BCL2-rearrangement**	
Chi-squared	0.4977	
p-value	0.48	
**Hazard ratios with 95% Confidence Interval**		
**Factor**	**0**	**1**
0	-	1.4605 (0.4831 to 4.4155)
1	0.6847 (0.2265 to 2.0701)	-

Table S1. Extranodal involvement.

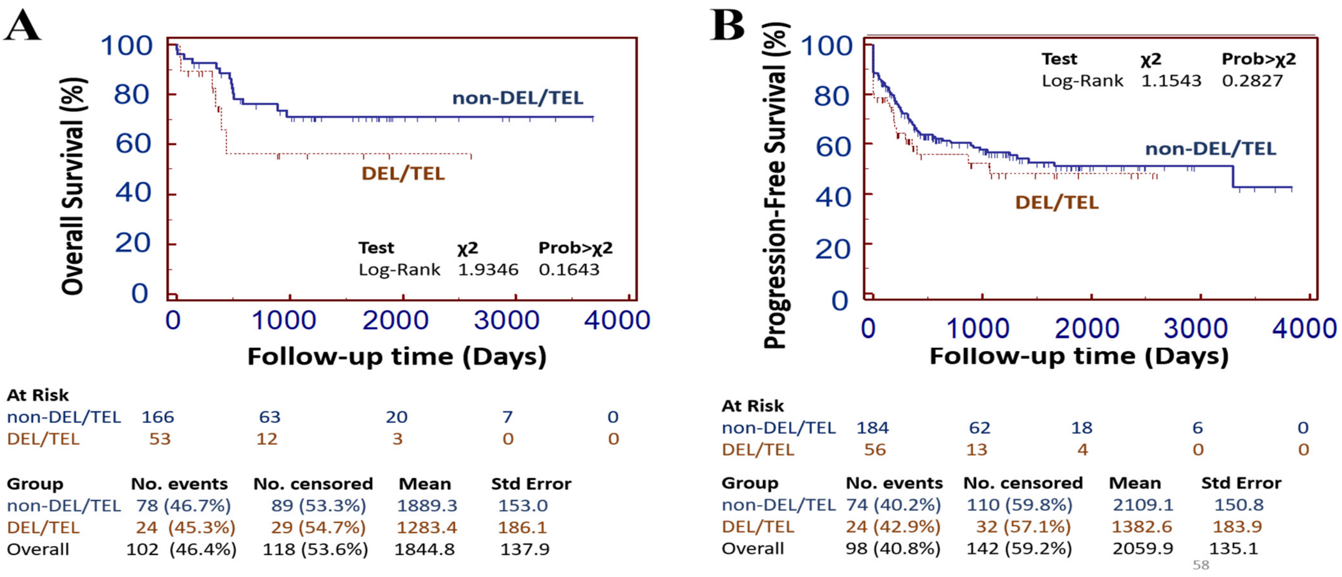

Figure S1. Multi-gene expression (DEL/TEL) confer poor prognosis. Kaplan-Meier curves showing the differential effect of DEL/TEL or non-DEL/TEL on the (**A**) overall or (**B**) progression-free survival of our DLBCL cohort (n = 282). DHL, double hit lymphoma; THL, triple hit lymphoma; DEL, double expressor lymphoma; TEL, triple expressor lymphoma; X^2, chi-square.

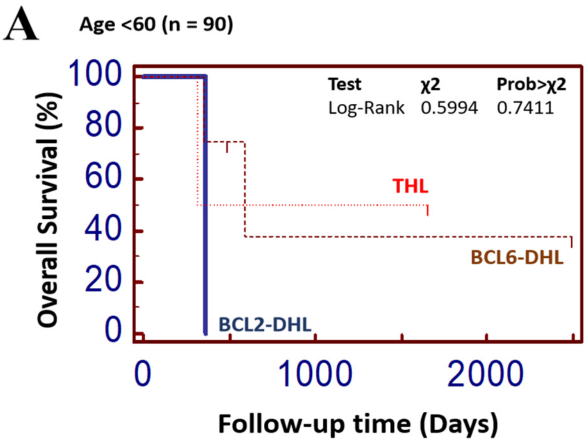

At Risk

BCL-DHL	1	0	0	0	0	0
BCL6-DHL	4	2	1	1	1	0
THL	2	1	1	1	0	0

Group	No. events	No. censored	Mean	Std Error
BCL2-DHL	1 (100.0%)	0 (0.0%)	358.8	0.0
BCL6-DHL	2 (50.0%)	2 (50.0%)	1243.6	567.7
THL	1 (50.0%)	1 (50.0%)	985.0	473.1
Overall	4 (57.1%)	3 (42.9%)	1208.8	410.6

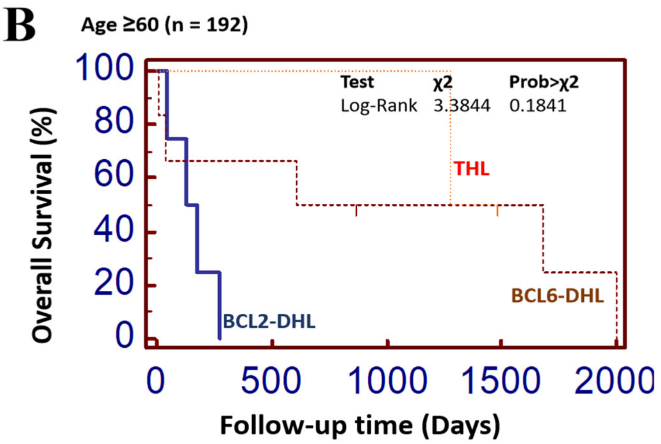

At Risk

BCL-DHL	4	0	0	0	0
BCL6-DHL	6	4	2	2	0
THL	2	2	2	0	0

Group	No. events	No. censored	Mean	Std Error
BCL2-DHL	4 (100.0%)	0 (0.0%)	156.8	47.7
BCL6-DHL	5 (83.3%)	1 (16.7%)	1028.3	386.0
THL	1 (50.0%)	1 (50.0%)	1381.0	74.2
Overall	10 (83.3%)	2 (16.7%)	814.3	240.8

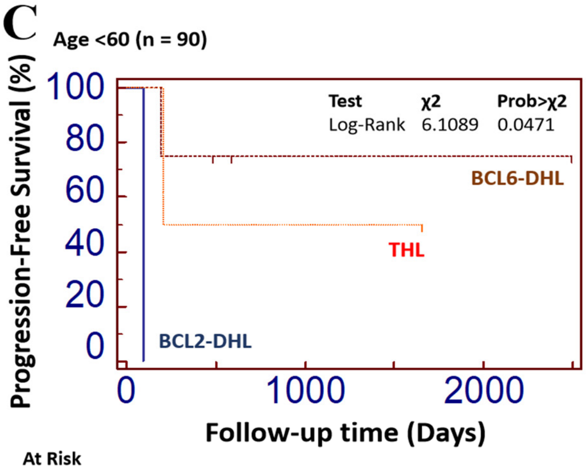

At Risk

BCL2-DHL	1	0	0	0	0	0
BCL6-DHL	4	2	1	1	1	0
THL	2	1	1	1	0	0

Group	No. events	No. censored	Mean	Std Error
BCL2-DHL	1 (100.0%)	0 (0.0%)	92.0	0.0
BCL6-DHL	1 (25.0%)	3 (75.0%)	1919.5	498.4
THL	1 (50.0%)	1 (50.0%)	928.5	513.0
Overall	3 (42.9%)	4 (57.1%)	1495.4	436.4

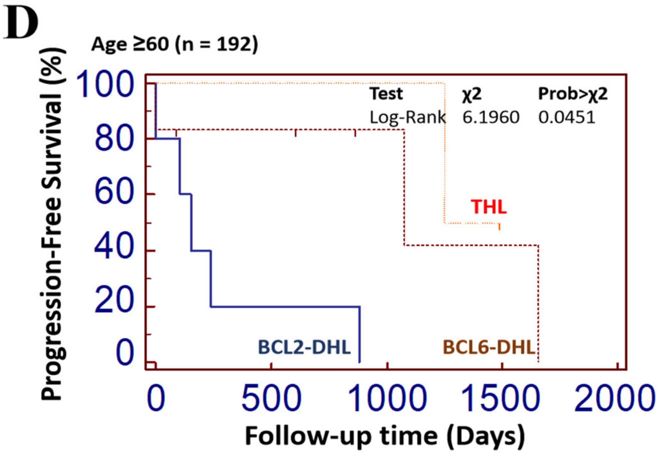

At Risk

BCL2-DHL	5	1	0	0	0
BCL6-DHL	6	4	2	1	0
THL	2	2	2	0	0

Group	No. events	No. censored	Mean	Std Error
BCL2-DHL	5 (100.0%)	0 (0.0%)	274.0	156.2
BCL6-DHL	3 (50.0%)	3 (50.0%)	1137.7	329.7
THL	1 (50.0%)	1 (50.0%)	1368.5	83.1
Overall	9 (69.2%)	4 (30.8%)	813.5	200.2

Figure S2. Age-adjusted survival analysis. Kaplan-Meier curves showing the differential effect of BCL2-DHL, BCL6-DHL or THL on the overall survival of our DLBCL cohort (n = 282) (**A**) younger than, or (**B**) older than 60 years. Kaplan-Meier curves showing the differential effect of BCL2-DHL, BCL6-DHL or THL on the Progression-free survival of our DLBCL cohort (n = 282) (**C**) younger than, or (**D**) older than 60 years. DHL, double hit lymphoma; THL, triple hit lymphoma; X^2, chi-square.
